# A Phylogeographical Analysis of the Beetle Pest Species *Callosobruchus chinensis* (Linnaeus, 1758) in China

**DOI:** 10.3390/insects13020145

**Published:** 2022-01-29

**Authors:** Fang Wang, Min Li, Haixia Zheng, Tian Dong, Xianhong Zhang

**Affiliations:** 1 College of Plant Protection, Shanxi Agricultural University, Jinzhong 030801, China; wangruifang24@163.com (F.W.); zhenghaixia722@163.com (H.Z.); 18234475698@163.com (T.D.); 2 Department of Biology, Taiyuan Normal University, Jinzhong 030619, China; limin12nk@163.com

**Keywords:** *Callosobruchus chinensis*, mitochondrial gene, phylogeography, the distribution modelling (SDM), least-cost path (LCP)

## Abstract

**Simple Summary:**

*Callosobruchus chinensis*, a stored product pest, is difficult to control. In the study, the goal was to explore the causes of the demographic history, dispersal path and genetic variations underlying the spatial and temporal distribution of *C. chinensis* in China. The phylogeography of *C. chinensis* was analyzed by distribution modelling (SDM) under six periods and the least-cost path (LCP) based on combined mitochondrial DNA. Our data showed that the geographical isolation of the genetic lineages and the distribution range of *C. chinensis* were restricted by climate in different times. The landscape structure had influence on the genetic differentiation of *C. chinensis*. Although the migration ability of *C. chinensis* is limited, the development of transportation and trade is helping the insect spread, along with the beans of its host.

**Abstract:**

*Callosobruchus chinensis* (Coleoptera Bruchidae), is a pest of different varieties of legumes. In this paper, a phylogeographical analysis of *C. chinensis* was conducted to provide knowledge for the prevention and control of *C. chinensis*. A total of 224 concatenated mitochondrial sequences were obtained from 273 individuals. Suitable habitat shifts were predicted by the distribution modelling (SDM). Phylogeny, genetic structure and population demographic history were analyzed using multiple software. Finally, the least-cost path (LCP) method was used to identify possible dispersal corridors and genetic connectivity. The SDM results suggested that the distribution of *C. chinensis* experienced expansion and contraction with changing climate. Spatial distribution of mtDNA haplotypes showed there was partial continuity among different geographical populations of *C. chinensis*, except for the Hohhot (Inner Mongolia) population. Bayesian skyline plots showed that the population had a recent expansion during 0.0125 Ma and 0.025 Ma. The expansion and divergent events were traced back to Quaternary glaciations. The LCP method confirmed that there were no clear dispersal routes. Our findings indicated that climatic cycles of the Pleistocene glaciations, unsuitable climate and geographic isolation played important roles in the genetic differentiation of *C. chinensis*. Human activities weaken the genetic differentiation between populations. With the change in climate, the suitable areas of *C. chinensis* will disperse greatly in the future.

## 1. Introduction

Phylogeography is the study of the evolutionary history of species, explaining the present and past distribution patterns of species [1,2,3]. Climatic conditions, geographic isolation, and human activities are factors that have become increasingly implicated in the genetic structure and population demographic history of multiple species [3,4,5,6].

Geographic isolation is essential for the genetic structure of populations. A population may be physically separated when its original habitat becomes divided by natural barriers (e.g., mountains and rivers). These barriers prevent gene flow and result in the genetic differentiation of isolated subpopulations [7,8]. Some studies have also demonstrated the importance of this in the phylogeography of various species. For example, the Mississippi River and the Appalachian Mountains in eastern North America caused genetic differentiation among populations of some taxa [9,10,11,12,13,14]. Geographic isolation has resulted in strong genetic differences between populations such as the caddisfly, in western and Eastern Europe [15]. China has a vast territory, a wide span of latitudes, and provides a wide diversity of climates. China’s geography is characterized by rivers and mountains, which prevents gene flow between populations, thereby promoting genetic divergence [9,16,17,18]. The Qinling, Daba and Taihang Mountains have been proven to be barriers for species’ genetic communication [19,20,21]. The rivers (i.e., Yangtze River, Yellow River, Huai River) also play an important role in genetic variation [21,22,23].

Climate conditions are closely related to the survival of insects. The Pleistocene glaciations have been recent climatic cycles, usually resulting in genetic variation, which further affect population demographics [7,24,25]. The last interglacial period (LIG, ~130–116 ka) is considered to have been warmer than the present, and its sea level was higher than present levels in most parts of the world [26,27,28,29]. The last glacial maximum (LGM) was about 22,000 years ago, with extreme dry and cold climatic conditions [30]. In LGM, large parts of the Northern Hemisphere were covered by ice sheets and sea levels dropped by an average of 120 m [31]. Many phylogeographical studies have focused on the LGM and LIG periods, and pre-LGM expansion and post-LGM expansion have been recorded for some species [32]. Compared to the early and late periods, the Mid-Holocene (MH, past 6000 years) was characterized by the greatest change in climatic conditions [33,34]. The mean annual temperature in the MH was 1–2 degrees Celsius higher than at present [35]. The Holocene climate drove the expansion of many species [32,36]. However, an analysis of the potential distribution of the herb *Cytisus oromediterraneus* at four periods (i.e., LIG, LGM, MH and Current), indicated a similar potential range during MH and the present periods [37].

*Callosobruchus chinensis* is widely distributed in China, breeding exclusively on many kinds of beans, such as kidney beans, cowpeas, lentils, chickpeas, broad beans and mung beans [38]. Due to its strong reproductive ability and the continued harm in seed storage, it causes considerable losses in quality and quantity [39,40]. *C. chinensis* may be derived from remaining populations on-site, it may be transmitted by flying or bean transportation, and it may also come from bean processing equipment [41]. Previous studies have shown that differences in geographical latitude and climatic conditions have resulted in differences in spawning, hatching rate, development duration and feeding habits among various populations of *C. chinensis* [42]. Therefore, the genetic structure of the *C. chinensis* population is likely to be affected by geography, climate and human activity [43,44].

In this study, our main goal was to estimate the importance of geographic barriers, climatic factors, geographic distance, and human intervention to the genetic structure and population demographic history of *C. chinensis*. It is difficult to directly observe pests’ population structure and long-distance migrations. Molecular markers therefore serve as a very useful tool to study these parameters [45]. In this paper, the predicted distribution of *C. chinensis* habitat at six periods was modelled. Phylogenetic and phylogeographical analyses, as well as the least-cost path (LCP), were conducted to determine possible structure-causing factors. This study provides dates on the mitochondrial gene data of the *C. chinensis* population samples, and therefore its genetics, in the main planting areas of mung bean. The study also provides knowledge for appropriate strategies in predicting pest infestations.

## 2. Materials and Methods

### 2.1. Specimen Collection

A total of 273 individuals of *C. chinensis* were collected from 22 mung bean planting sites in China from 2017 to 2019 (Supplementary Table S1 and Figure S1). All samples were preserved in 95% ethanol and stored at −20 °C before DNA extraction.

### 2.2. DNA Extraction, Amplification, Sequencing and Sequence Editing

Total genomic DNA was extracted from the thorax muscle of *C. chinensis*, using the Biospin Insect Genomic DNA Extraction Kit (BIOER, Hangzhou, China). The mitochondrial fragments of cytochrome oxidase subunit I (*COI*), cytochrome oxidase subunit II (*COII*), cytochrome b (*Cyt-b*) and 12S ribosomal RNA (12S rRNA) were amplified using PCR Master Mix. PCR-specific primers (Table 1) were designed based on the *C. chinensis* sequences KY856744 from GenBank. The PCR amplification procedures were as follows: 94 °C for 3 min, 35 cycles of 94 °C for 30 s, 56 or 62 °C for 20 s, 72 °C for 30 s, and a final extension at 72 °C for 10 min. The PCR products were examined using 1% agarose gels with ethidium bromide following electrophoresis. The products of *COI*, *COII*, *Cyt-b* and 12S rRNA were sent to Shanghai Invitrogen for sequencing in both directions.

BioEdit 7.1.7 [46] was applied in visual proofreading. Multiple sequences check, editing and alignment were performed using the MEGA X software. [47]. The mtDNA (*COI*, *COII* and *Cyt-b*) reading frames were checked in MEGA X, which revealed no evidence of putative nuclear pseudogenes [48,49] in the dataset.

### 2.3. Ecological Niche Modelling

This study used data on 80 occurrence points of *C. chinensis* in China, including 22 field collection points and 58 data points found by consulting related published literature and the GBIF (Global Biodiversity Information Facility) (Supplementary Table S2 and Figure S2). Species distribution modelling (SDM) was performed to evaluate the potential distribution of *C. chinensis* throughout the late Quaternary, current and future periods. The variables of 19 climate factors (Supplementary Table S3) for current conditions, for future conditions, and for three time slices of the late Quaternary, were retrieved from the WorldClim database version 1.4 (http://www.worldclim.org, accessed on 23 October 2021) [50]: (1) Last Inter-Glacial (LIG), about 120,000 to 140,000 years ago; (2) Last Glacial Maximum (LGM), about 22,000 years ago; (3) Mid-Holocene (MH), about 6000 years ago; (4) Current, 1975 (average value from 1960–1990); (5) Future, 2050 (average value from 2041–2060); (6) Future, 2070 (average value from 2060–2080). Except for the spatial resolution of the raster data of the last glacial maximum of 2.5 arc minutes (5 km × 5 km), that of the other five periods were all 30 arc seconds (1 km × 1 km). Pearson correlation analysis was used to test the correlation between the 19 climate factors in ArcGIS 10.4.1 (Esri, Redlands, CA, USA) and IBM SPSS Statistics 20.0 for Windows (IBM Corp, Armonk, NY, USA). When the correlation coefficient of two factors was greater than 0.85, one of them was omitted. Finally, nine factors were selected for suitability analysis: annual mean temperature (BIO1); mean diurnal range (BIO2); isothermality (BIO3); temperature seasonality (BIO4); mean temperature of wettest quarter (BIO8); mean temperature of warmest quarter (BIO10); mean temperature of warmest quarter (BIO13); precipitation of driest month (BIO14); and precipitation of warmest quarter (BIO18). The maximum entropy modeling of species geographic distributions was analyzed in MaxEnt 3.4.1 software [51]. The parameters of the current periods in the model were set as follows: the proportion of test data was 25%, the regulation multiplier was 1, the maximum iterations were 1000, the convergence threshold was 0.00001, and it was run 10 times to achieve the best result. At the same time, area under the curve (AUC) of the receiver operating characteristic (ROC) was analyzed and calculated. The AUC values were higher than 0.8 of all ten runs, indicating the better and reliable predictions of the model. The distribution model under current conditions was projected to the LIG, LGM, MH, 2050 and 2070. Finally, the six habitat suitability layers were loaded in ArcGIS 10.4.1. Then, the data of the six periods were used to perform a pairwise comparison of the binary SDMs to predict the distributional changes of *C. chinensis* between two adjacent time periods (from LIG to LGM, from LGM to MH, from MH to Current, from Current to 2050, from 2050 to 2070) by SDMtoolbox 2.4 [52].

### 2.4. Genetic Polymorphism Analysis and Isolation by Distance

The number of segregating sites (S), number and distribution of haplotypes (Hap), haplotype diversity (Hd), nucleotide diversity (Pi), standard deviation of haplotype diversity; standard deviation of Pi, and mismatch distributions of pairwise nucleotide differences were analyzed using DnaSP 5.0 [53]. Recent expansion led to unimodal mismatch distributions in populations. Arlequin 3.5 [54] was used to calculate molecular Pairwise Φ_ST_ (Phi_ST_), Tajima’s D and Fu’s Fs. The values of Tajima’s D and Fu’s Fs were significantly negative, which indicated recent demographic expansions for species. To test a hypothesis of isolation by distance (IBD), we used a Mantel test which was calculated with the matrix of genetic distance (Φ_ST_/(1 − Φ_ST_)) versus the matrix of geographical distance (ln km) in the GenAlEx 6.501 software [55].

### 2.5. Phylogeographical Analysis

PopART 1.7 [56] was used to construct a median-joining network (MJ) with default settings. Two methods were applied to study genetic structure and potential geographic barriers of *C. chinensis*, using the concatenated mitochondrial genes. BAPS 6.0 [57] was used to calculate population structure for spatial clustering. The K value corresponding to log (ml) value was used as the optimal value of the population space grouping. Monmonier’s maximum difference algorithm in BARRIER 2.2 [58] was applied to identify major biogeographical barriers in population samples.

### 2.6. Reconstructions of Divergence Time and Historical Demography

To investigate the lineage divergence of *C. chinensis*, we reconstructed an intraspecific phylogeny based on concatenated mitochondrial haplotypes, with a set make up of a constant population size coalescent model, a relaxed uncorrelated lognormal molecular clock and a GTR (General Time Reversible) substitution model in BEAST 2.6.3 [59]. Since there were no fossil records and no clear biogeographic events for the calibration of the trees, we employed an estimated rate of 0.0115 substitutions/site/MY and a standard deviation of 0.0005 [60,61,62]. This rate is the standard arthropod rate [63], which may be different for *C. chinensis*, but was similar to rates obtained based on fossil and biogeographic events in some Coleoptera for the combined mitochondrial genes [64,65]. Six independent MCMC analyses were run for 100,000,000 generations. Tracer 1.7 [66] was used to confirm stationarity, and then TreeAnnotator 2.6 [67] was used to construct a maximum credibility tree. A Bayesian skyline plot (BSP) was generated in BEAST 2.6.3 and reconstructed in Tracer 1.7. When the ESS (effective sample size) value was larger than 200, the result was available.

### 2.7. Visualization of Dispersal Corridors

SDMtoolbox 2.2 in ArcGIS 10.4.1 was used to create the least-cost paths of *C. chinensis*. This involved, first, creating a friction layer by inverting SDM, and second, using shared haplotype data of mitochondrial genes (*COI* + *COII* + *Cyt-b* + 12S) of the *C. chinensis* population samples to calculate the cost of the diffusion path between geographic locations of different population samples. Third, we set the classification values of the lowest cost values 0.05, 0.02 and 0.01, which were shown in different colors in the diffusion connectivity layer. Finally, we summarized and standardized all the reclassified paired corridor layers, and determined the scattered corridors of *C. chinensis* in a clear landscape.

## 3. Results

### 3.1. MaxEnt Model Evaluation

Evaluations of the MaxEnt model showed that, the AUC values of all the training samples and test samples were higher than 0.8 under the current climatic conditions. The best AUC value of the training date and test date were 0.900 and 0.857, respectively, indicating that the prediction result on potential distribution of *C. chinensis* was accurate and reliable (Supplementary Figure S3).

### 3.2. Prediction of the Range and Change in Habitat Suitability of C. chinensis

**Figure 1:**
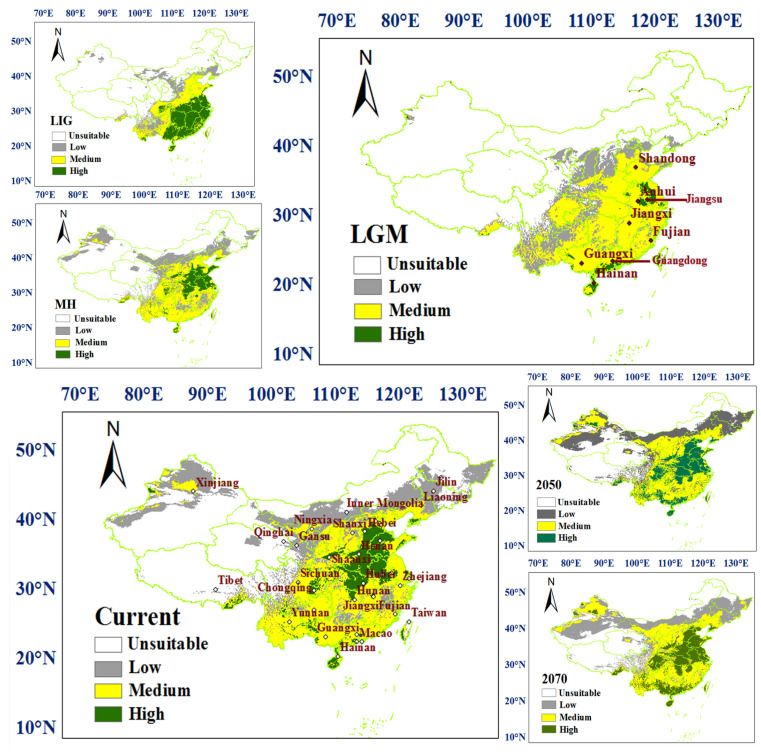
Distribution of habitat suitability for *Callosobruchus* *chinensis* in China under six different climatic conditions, inferred using species distribution modelling (SDM). The different color indicates the degree of suitability.

**Figure 2:**
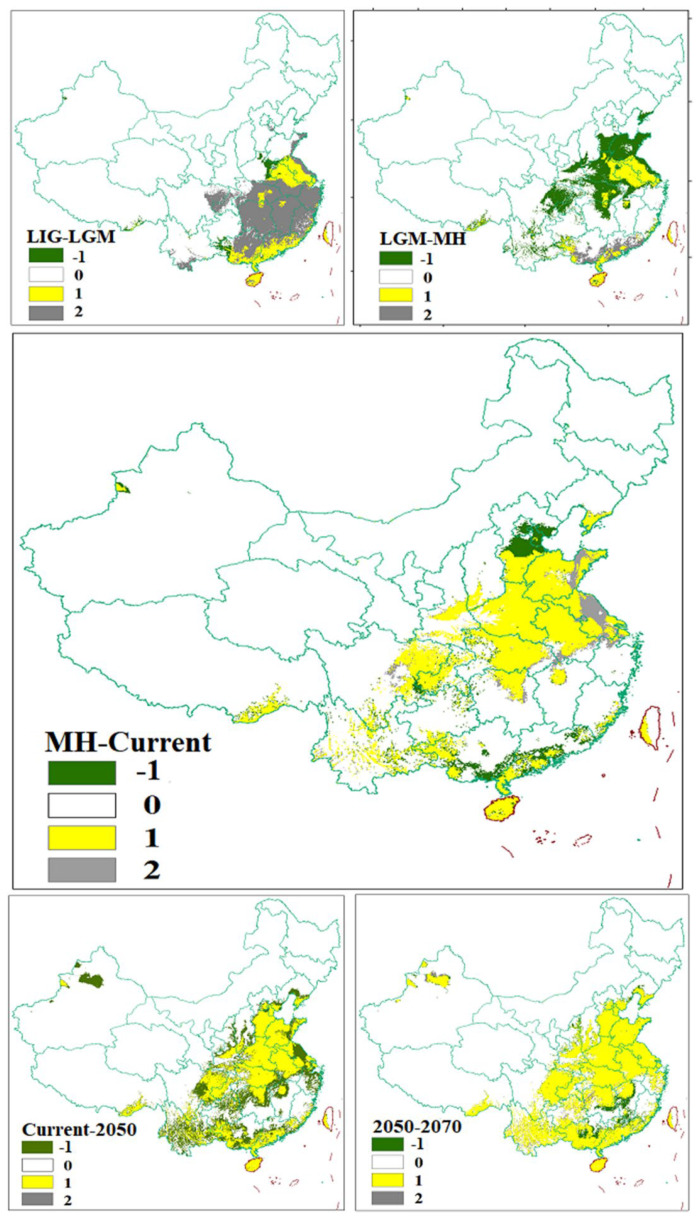
Predicted changes in the distribution areas of suitable habitat for *Callosobruchus* *chinensis* in China between two adjacent time periods. “-1” represents the expansion areas, which is the green region in figures; “0” represents the areas where the species did not exist, which is the white region in figures; “1” represents the areas where the distribution had not changed, which is the yellow region in figures; “2” represents the areas where the distribution was decreased, and this is the gray region in figures.

The prediction results of the habitat suitability of *C. chinensis* in China under six different periods are shown in Figure 1. From LIG to 2080, this beetle had experienced population shrinkage and expansion with environmental changes in different periods. For high suitability regions, the distribution of *C. chinensis* was mainly in the southeast of China under LIG scenario; these showed a significant reduction under the LGM scenario. The distribution of *C. chinensis* was mainly in Shandong, Anhui, Henan and Jiangsu from LGM to 2070, and the scope was constantly increasing. The low and medium suitability regions were enlarged successively under the six periods in North China. The changes in habitat suitability of *C. chinensis* are shown in Figure 2. Between the LIG and LGM periods, the habitat suitability had a significant contraction in the southeast of China. Comparison of the LGM and MH periods showed that the habitat suitability had a significant expansion in the middle-east of China, and then, around the original sites. Under LGM climatic conditions, only the east (parts of Jiangsu and Anhui) and the south of China (the coastal areas of Hainan, Guangdong and Guangxi) provided potential refuges for *C. chinensis*.

### 3.3. Genetic Polymorphism Analysis

In this study, mitochondrial sequence dates were obtained from 273 individual *C. chinensis* beetles, which provided a data matrix of 2803 bp, comprising 12S (549 bp), *COI* (845 bp), *COII* (600 bp) and *Cyt-b* (809 bp) gene fragments. A total of 56 haplotypes were identified based on the concatenated mitochondrial genes. The concatenated mitochondrial haplotype diversity (Hd) ranged from 0.378 to 1, only the values of HB2 and GZ were less than 0.5, and especially the value of GZ was much smaller than the others. The nucleotide diversities ranged from 0.00021 to 0.00084. The values of SCW, SYQ, SD2, HL, HB2, TJ and GZ were less than 0.0005; however, the values of SJT and JX were higher than other populations (Table 2). Fifteen of the 56 haplotypes were found in at least two of 22 populations and the remaining 41 haplotypes were found only in one population. Hap-3 and Hap-4 were present in the largest number and proportion of population samples, with Hap-3 being more prevalent in populations in the south of China, and Hap-4 being more frequent in the northern regions, such as the Shanxi populations.

### 3.4. Phylogenetic and Phylogeographical Analyses

**Figure 3:**
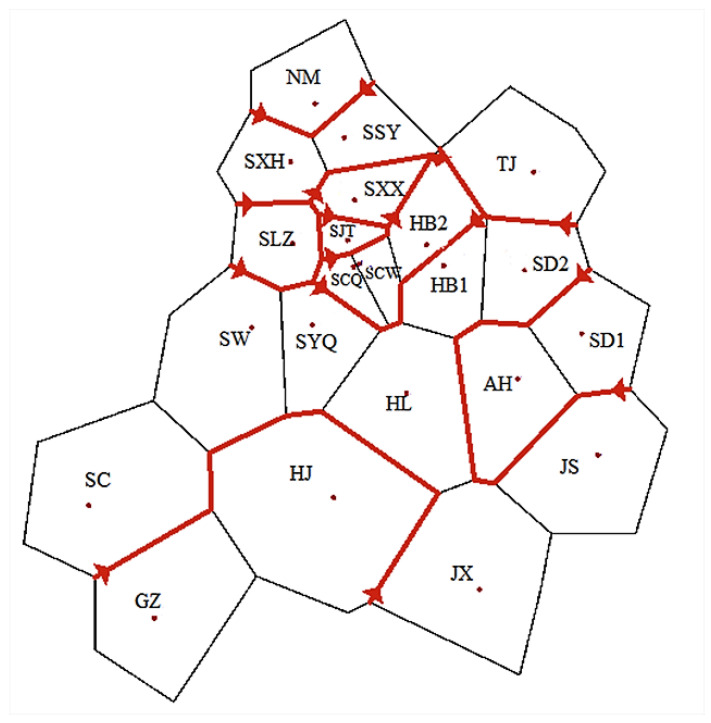
Genetic boundaries detected by barrier analysis based on Monmonier’s algorithm. Black lines show Voronoi tessellation and red lines labeled areas indicate genetic discontinuities. The red lines with arrows at both ends indicate the eight main boundaries.

**Figure 4:**
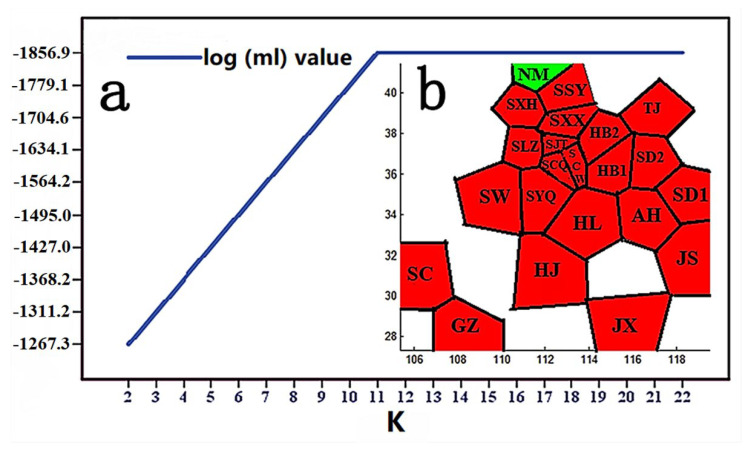
The approach for identifying the population genetic structure of *Callosobruchus chinensis* in China based on concatenated mitochondrial genes. (**a**) When K was 2, the log (ml) value was used as the optimal value of the population space grouping; (**b**) BAPS clustering.

**Figure 5:**
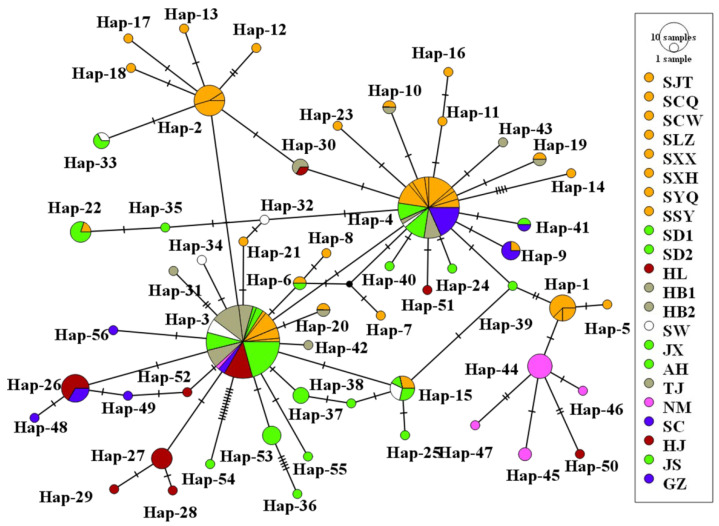
MJ analysis of the network of *Callosobruchus chinensis* based on concatenated mitochondrial haplotypes. The circles represent different haplotypes with their proportional to the number of individuals. The colors represent different populations. The short line segments indicate mutated positions between haplotypes.

The BARRIER analysis showed eight boundaries (Figure 3). BAPS analysis showed that the NM population had high genetic differentiation (Figure 4). Network analyses showed there were obviously two clades on the whole, but with lots of subdivisions (Figure 5). The haplotype networks showed a star-like topology typical of recent population expansion, with two common haplotypes (Hap-3 and Hap-4) and many local rare ones.

### 3.5. Isolation by Distance

Mantel tests detected no positive correlations among the 22 populations of China, denoted by pairwise Φ_ST_/(1 − Φ_ST_) and geographical distances between populations for concatenated mitochondrial (12S + *COI* + *COII* + *Cytb*) genes (r = 0.069, *p* = 0.310) (Supplementary Figure S4).

### 3.6. Intraspecific Divergence Time and Historical Demographic Reconstruction

**Figure 6:**
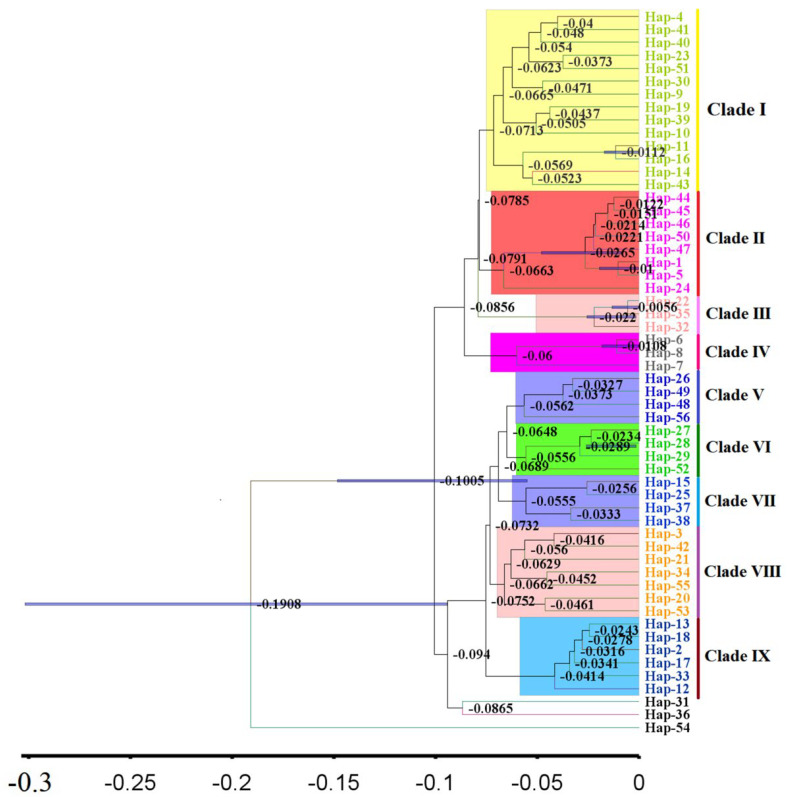
The mitochondrial estimation of population divergence time of *Callosobruchus chinensis* based on BEAST.

**Figure 7:**
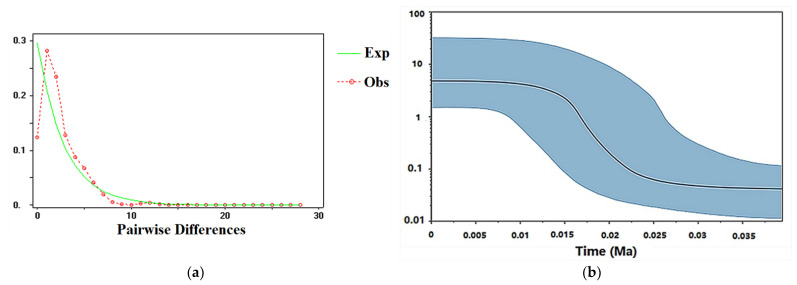
(**a**) Mismatch distribution curves of the entire samples for *Callosobruchus chinensis*. Observed and expected mismatch distribution are the red and green lines, respectively. (**b**) Historical demographic trends of the entire samples of *Callosobruchus chinensis* in China were implemented by Bayesian skyline plots (BSP), using an estimated rate of 0.0115 substitutions/site/MY and a standard deviation of 0.0005. The historical time (Ma) is displayed on the x-axis, and the effective population size is shown on the y-axis. The black solid line represents the median of population size, and blue areas represent 95% HPD.

The results from the BEAST analysis showed that the two big branches began to diversify at 0.1005 Ma (Figure 6). The first one had four clades, named Clade I, Clade II, Clade III and Clade IV, and the divergence of the four clades occurred at 0.0713, 0.0785, 0.0791 and 0.0856 Ma, respectively. The second branch had five clades, called Clade V, Clade VI, Clade VII, Clade VIII and Clade IX, and the divergence of five clades occurred at 0.0562, 0.0648, 0.0689, 0.0732 and 0.0752 Ma, respectively (Figure 6). The divergence time of the populations were mainly between 0.05 Ma and 0.11 Ma. The unimodal mismatch distributions, and the significant negative values of Tajima’s D and Fu’s Fs, indicated that *C. chinensis* populations have experienced a recent expansion (Table 2 and Figure 7a). The Bayesian skyline plots (BSP) indicated the demographic history of *C. chinensis* in China (Figure 7b). It also showed that the population of *C. chinensis* may have experienced three main periods: the first stage was a prolonged phase of demographic stability; the second stage was a recent population expansion during 0.0125 Ma and 0.025 Ma; the third stage was a prolonged phase of demographic stability after 0.0125 Ma (Figure 7b).

### 3.7. Visualization of Dispersal Corridors and Gene Flow Estimation

**Figure 8:**
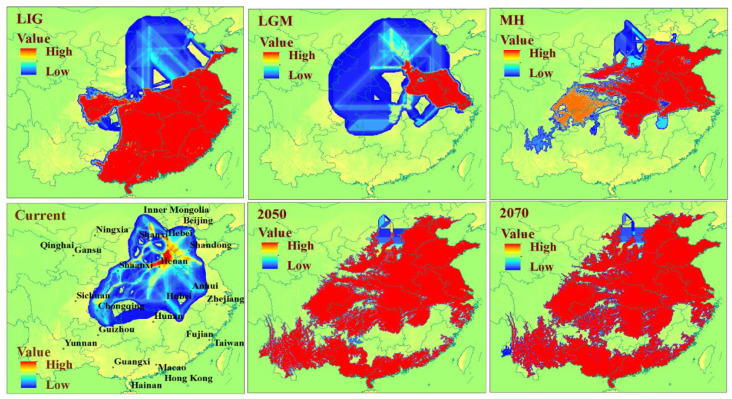
Potential dispersal routes of *Callosobruchus chinensis* in China across six time periods based on mitochondrial markers. Different colors represent different diffusion path costs, from red to yellow to blue, diffusion costs gradually increase.

The LCP analysis of *C. chinensis* based on shared haplotypes showed the putative dispersal corridors for the six periods (Figure 8). Change in the climate in the six periods has resulted in drastic changes in the genetic connectivity of the *C. chinensis* population (Figure 8). In the LIG period, high genetic connectivity occurred in the southeast of China. In the LGM period, the scope enormously shrank, and the high genetic connectivity of *C. chinensis* mainly occurred in Jiangsu, Anhui and Henan. In the MH period, there was a huge expansion around the sites of Hebei, Shandong, Shaanxi, Jiangsu, Anhui, Henan and Hunan. In the present period, the high genetic connectivity of *C. chinensis* mainly occurred in the region of Shanxi-Henan-Hebei. Finally, in the future of 2050 and 2070, the landscape connectivity revealed that *C. chinensis* will disperse widely in north China (Beijing, Tianjin, Hebei, Shanxi), east China (Shanghai, Jiangsu, Zhejiang, Jiangxi, Anhui, Fujian and Shandong), central South China (Henan, Hubei, Hunan, Guangdong, Guangxi and Hainan), southwest China (Chongqing, Sichuan, Guizhou and Yunnan) and Shaanxi.

## 4. Discussion

Molecular markers have proven to be very useful in solving the genetic structure of beetle populations [68]. In this study, we collected samples of *C. chinensis* from 22 geographic sites in China. These *C. chinensis* beetles were analyzed for their patterns based on concatenated mitochondrial sequences (*COI*, *COII*, *Cyt-b*, and 12S rRNA). Our results showed that the genetic diversity (Hd) was seemingly high among populations, which was consistent with other Coleopterans, i.e., *Cosmopolites sordidus* (Germar 1824), *Sitophilus zeamais* (Motsch.), *Callosobruchus* *maculatus* (Fabr., 1775) [68,69,70]. The high haplotype diversity (Hd > 0.5) and low nucleotide diversity (Pi < 0.005) indicated that the *C. chinensis* population had experienced a bottleneck effect. The results of multiple analysis (unimodal mismatch distributions, significant negative values of Tajima’s D and Fu’s Fs, scattered network and prediction of habitat suitability) indicated that *C. chinensis* then underwent expansion and genetic variation. At the same time, it also showed that *C. chinensis* had high environmental adaptability [44].

In China, the influencing factors on the phylogeographic structure of many species, such as *Hyalessa maculaticollis* (Motschulsky, 1866), *Camaena cicatricosa* (Müller, 1774), *Metrocoris sichuanensis* (Chen and Nieser, 1993) and *Scythropus yasumatsui* Kono et Morimoto, have been studied [7,45,71,72]. Genetic variation of agricultural insects can be affected by several factors, including geographical barriers, climate factors, climate oscillations of the Quaternary, human activities, etc. [42].

Previous phylgeographic studies indicated that mountains have acted as geographical barriers to drive speciation in geographically-isolated populations [73,74,75,76]. For example, the genetic divergence exhibited by many insects, e.g., *Leptinotarsa Decemlineata* (Say), *Carabus solieri*, and *Epipyropidae*, is due to geographical barriers [77,78,79]. In this study, the haplotypes among population samples demonstrated that most haplotypes only exist in a specific geographic area, and Hap-4 was more frequently in Shanxi populations, suggesting that there was a degree of genetic differentiation among different locations. The result from the BARRIER analysis showed there were obstacles among Shanxi populations (SLZ, SJT, SXX, SCQ, SCW, etc.) and other populations, which indicated gene exchange among them was limited. Shanxi, which lies on the Loess Plateau, west of the Taihang Mountains, locates on the second step of Chinese topography. On the Loess Plateau, Shanxi is dominated by a high gully density landscape [80], which become natural barriers for species in migration. The results of SDM (Figure 1 and Figure 2) showed that ecological niches in Shanxi were suitable for the survival of *C. chinensis*, which suggested that climate conditions are not an effective biogeographic barrier for Shanxi populations. In this study, these results indicated that the Taihang Mountains and Loess Plateau should be natural barriers of Shanxi (SLZ, SJT, SXX, SCQ, SCW, etc.) to restrict gene exchange. Their function of geographical isolation has been found in other species, e.g., *S. yasumatsui* [72]. Moreover, the Taihang mountains have acted as a geographical barrier in *Sitodiplosis mosellana* (Géhin) (SM) [81].

Climatic conditions have been considered to be a fundamental factor underlying population differentiation [73,82,83,84,85]. For insects, climatic conditions play an even more important role in shaping the population structure of insects such as *Stomoxys calcitrans* (L.) and *Stomoxys niger niger* Macquart [86]. This is especially so for climatic conditions unfavorable to insect development [86,87,88]. In the case of *C. chinensis*, the BAPS and BARRIER analysis indicated that the NM population had high genetic divergence. The NM population samples was collected from Hohhot, a region characterized by long and cold winters. The isolation by distance (IBD) analysis showed no relationship between genetic differentiation and geographic distances among the 22 populations. This indicates that genetic isolation may have been caused by other factors than geographical distance between population samples. Indeed, climatic conditions of a species’ habitat are often barriers to dispersal. These results suggest that climate played an important role in preventing migration between populations, which were consistent with the prediction results of the habitat suitability of *C. chinensis* in China. Temperature is the primary variable affecting the distribution and population dynamics of species [89,90]. BIO1, BIO8 and BIO10 were considered to be the important factors affecting the habitat adaptation of *C. chinensis* (Supplementary Figure S5), which further confirmed the influence of temperature on genetic variation of *C. chinensis* populations. Rising temperatures are the most remarkable element of climate change, leading to the increase in insect pests [91,92]. Researchers have shown that there is a trend of gradual expansion of some insects to the north of China [8,33]. According to the results of this work, the suitable distribution areas for *C. chinensis* will significantly increase not only in northern China but also in other regions, and there will be a tendency to spread across the country. Consequently, the management of *C. chinensis* will become even more difficult.

It has been demonstrated that Quaternary climate change has had a huge influence on many species’ distributions and population structures [4,15]. Our analyses (i.e., mismatch distributions, Tajima’s D, Fu’s Fs, network of haplotypes, BEAST analysis and BSP) all indicated a recent expansion, and also showed that the divergence time of *C. chinensis* populations mainly occurred in Quaternary glaciations. The habitat ranges of *C. chinensis* populations, from the LIG to the present periods, have experienced demographic expansions (pre-LGM expansion and post-LGM expansion) and a contraction. The expansions agree with historical events, corresponding to the warming climate under the LIG and MH periods. Pre-LGM expansion has also been found in other plants and animals [27,93,94]. A rapid expansion in *C. chinensis* occurred during the MH period, which was also found in *C. maculatus* [70]. In this study, it was concluded that Quaternary climate fluctuation affected the distribution pattern and genetic diversity of *C. chinensis*. The theory says that the separation and expansion of populations with climate fluctuations lead to gene exchange via lineage admixture [4,95,96]. During ice ages, *C. chinensis* may retreat to shelters in eastern and southern China. After climate warming, these surviving *C. chinensis* populations are likely to expand rapidly and trigger gene flow between regions. Amusingly, the haplotypes distribution and Barrie analysis showed that AH population samples had genetic differentiation with adjacent populations and a higher value of Hd, which are also the potential refuges for *C. chinensis*. Moreover, the values of Hd and Pi of the GZ population were the smallest of all the populations. The location of the GZ population was far from the two refuges. Therefore, it is speculated that the pattern of genetic diversity of the GZ population may be largely due to the founder effect. The haplotypes distribution was related to the suitable distribution of *C. chinensis* during the late Quaternary, which confirmed the profound influence of changing climate on the present pattern of this weevil. The potential distribution of *C. chinensis* throughout the late Quaternary supported the hypotheses that the origin and dispersal of *C. chinensis* derived from South China and then spread from south to north. Similar patterns of origin and spread have been reported in other pests such as *Carposina sasakii* Matsumura and *Grapholita molesta* (Busck, 1916) [42,97].

Finally, human activities promote the long-distance dispersal of insects, and facilitate gene flow to weaken the genetic differentiation between populations [98,99,100]. The results (the haplotype network, BAPS analysis and BARRIER analysis) showed the Hap-3 and Hap-4 haplotypes shared by some populations, and many populations were genetically related closely, which revealed most of the populations may come from common origins or freely mating among populations. China has a vast territory with complicated topography and climate, which have hindered gene flow among *C. chinensis* populations. However, *C. chinensis* is highly migratory during a series of production, processing and commodity circulation processes, such as seed cultivation, harvesting, storage and transportation [41,44,100,101]. Therefore, there was frequent gene flow among different geographical populations, which reduced the genetic divergence among populations but enhanced the genetic diversity within populations [44]. As anthropogenic interventions in nature become stronger and stronger, the influencing factors of population structure are also changing. With the use of various pesticides, and the transport and trade of legumes, the genetic population structure of *C. chinensis* is increasingly influenced by anthropogenic intervention.

*C. chinensis* is widely distributed in China, and is an important and harmful beetle. In the future, global temperatures will be higher than they are now, due to the greenhouse effect. In recent years, some works have increasingly forecasted species’ distribution patterns under a future climate, which showed there is a trend of gradual expansion of some insects to the north of China [33,72]. The predication of suitable areas under future climatic conditions indicated that *C. chinensis* will spread widely in China. The survival rate of *C. chinensis* may increase in winter, and the number of generations of *C. chinensis* may increase. The prevention and control of this beetle is imperative. Several methods of analysis (DNA markes, MaxEnt model and LCP) have been employed to predict high suitability areas (Shandong, Hebei, Shanxi, Henan, Anhui, Jiangsu and Shaanxi) and a high genetic connectivity region (Shanxi-Henan-Hebei), which need to be monitored. Given the evidence from this study that there is potential gene flow between geographic populations due to human factors, it is important to ensure that there are no *C. chinensis* (e.g., larva and adult) before the bean seeds are transported and traded.

## Figures and Tables

**Table 1:** Primers’ sequences for amplification of four mitochondrial genes (*COI*, *COII*, *Cyt-b* and 12S rRNA) for *Callosobruchus chinensis*.

Gene	Primers	Annealing Temperature	Length (bp)
*COI*	F: AATAAATGATTATTTTCCACTAATCATAAAGACATCGGGA	56 °C	1533
	R: TTAATTTGTTAGTAGGGGTAATTCGGAGTATCTATG		
*COII*	F: ATTTTTACTTGAAAAACAATTCTTCTTCAAGAC	62 °C	688
	R: AAATTTTGATTATTTTAGAAATTCATTTAATAAAATAATTAGGAGT		
*Cyt-b*	F: ATGAAAATAAATTTTCGAAAAACCCACC	56 °C	1140
	R: TTAGTGGTAAATGATTTTATCTCATATTTTGTATAAAATTGA		
12S rRNA	F: AAAAAATTTTATTTTGGTTATTTAATTAGATTTTTCTTGGT	62 °C	752
	R: GTCTTTCTAGGCACACTTTCCAG		

**Table 2:** Nucleotide polymorphisms among 2803 bp of mtDNA sequence from *Callosobruchus chinensis* beetles in population samples across China.

Population Code	N	Hap	S	Hd ± SD	Pi ± SD	Tajima’s D	Fu’s Fs
SJT	10	Hap-1(5), Hap-2(1), Hap-3(1), Hap-4(2), Hap-5(1);	6	0.756 ± 0.130	0.00084 ± 0.00017	0.45768	−0.23033
SCQ	10	Hap-3(3), Hap-4(2), Hap-6(1), Hap-7(1), Hap-8(1);	4	0.857 ± 0.108	0.00056 ± 0.00013	0.08124	−1.69431
SCW	14	Hap-4(1), Hap-9(1), Hap-10(1);	2	1.000 ± 0.272	0.00048 ± 0.00016	0	−1.2164
SLZ	10	Hap-4(6), Hap-11(1), Hap-12(1), Hap-13(1), Hap-14(1);	10	0.667 ± 0.163	0.00082 ± 0.00023	−1.53448	−0.27358
SXX	10	Hap-2(5), Hap-4(1), Hap-15(2), Hap-16(1), Hap-17(1);	6	0.756 ± 0.130	0.00066 ± 0.00017	−0.53927	−0.78721
SXH	10	Hap-1(1), Hap-2(5), Hap-4(3), Hap-18(1);	6	0.711 ± 0.117	0.00067 ± 0.00019	−0.49593	0.44029
SYQ	10	Hap-3(5), Hap-4(1), Hap-19(1), Hap-20(1);	3	0.643 ± 0.184	0.00033 ± 0.00012	−0.81246	−1.38724
SSY	10	Hap-1(2), Hap-3(1), Hap-4(5), Hap-21(1), Hap-22(1), Hap-23(1);	8	0.800 ± 0.114	0.00078 ± 0.00019	−0.8324	−1.33144
SD1	10	Hap-3(2), Hap-4(4), Hap-22(4), Hap-24(1);	5	0.764 ± 0.083	0.00073 ± 0.00011	0.73905	0.80985
SD2	10	Hap-3(1), Hap-15(1), Hap-25(1);	2	1.000 ± 0.272	0.00048 ± 0.00016	0	−1.2164
HL	13	Hap-26(6), Hap-27(5), Hap-28(1), Hap-29(1);	4	0.679 ± 0.089	0.00049 ± 0.00007	0.25198	0.19892
HB1	12	Hap-3(4), Hap-10(1), Hap-19(1), Hap-30(2);	4	0.750 ± 0.139	0.00054 ± 0.00010	−0.12075	−0.42156
HB2	17	Hap-3(8), Hap-4(1), Hap-20(1), Hap-31(1);	4	0.491 ± 0.175	0.00026 ± 0.00011	−1.71166	−1.4146
SW	14	Hap-3(4), Hap-4(1), Hap-15(2), Hap-32(1), Hap33(1), Hap34(1);	6	0.844 ± 0.103	0.00054 ± 0.00013	−1.18946	−2.60454
JX	16	Hap-3(5), Hap-6(1), Hap-15(2), Hap-35(1), Hap-36(1);	11	0.756 ± 0.130	0.00084 ± 0.00034	−1.76515	−0.23033
AH	11	Hap-4(5), Hap-37(3), Hap-38(1), Hap-39(1), Hap-40(1), Hap-41(1);	5	0.803 ± 0.096	0.00057 ± 0.00010	−0.11051	−1.92425
TJ	12	Hap-3(5), Hap-4(4), Hap-42(1), Hap-43(1);	3	0.709 ± 0.099	0.00032 ± 0.00007	−0.38482	−0.93979
NM	12	Hap-3(1), Hap-44(7), Hap-45(2), Hap-46(1), Hap-47(1);	9	0.667 ± 0.141	0.00058 ± 0.00024	−1.83035	−0.69537
SC	11	Hap-3(2), Hap-9(3), Hap-26(3), Hap-48(1), Hap-49(1);	5	0.844 ± 0.080	0.00067 ± 0.00011	0.27556	−0.73268
HJ	23	Hap-3(8), Hap-30(1), Hap-50(1), Hap-51(1), Hap-52(1);	7	0.576 ± 0.163	0.00055 ± 0.00022	−1.3042	−0.83145
JS	14	Hap-3(13), Hap-33(2), Hap-53(4), Hap-54(1), Hap-55(1);	14	0.595 ± 0.108	0.00065 ± 0.00033	−2.04699	0.51876
GZ	14	Hap-4(8), Hap-41(1), Hap-56(1);	3	0.378 ± 0.181	0.00021 ± 0.00012	−1.56222	−0.45861
ALL	224		58	0.876 ± 0.016	0.00085 ± 0.00006	−2.24496	−26.7717

N: number of sequences of different geographic populations used in this study; S, number of segregating sites; Hap, haplotype distribution; Hd ± SD, haplotype diversity ± standard deviation of haplotype diversity; Pi ± SD, nucleotide diversity ± standard deviation of Pi.

## Data Availability

The mitochondrial sequences of cytochrome oxidase subunit I (*COI*), cytochrome oxidase subunit II (*COII*), cytochrome b (*Cyt-b*) and 12S ribosomal RNA (12S rRNA) have been uploaded in GenBank under accessions. MW249186–MW250183.

## Supplementary Materials

The following supporting information can be downloaded at: https://www.mdpi.com/article/10.3390/insects13020145/s1, Figure S1: Occurrence Data from field collection; Figure S2: Data on 80 occurrence points of *Callosobruchus chinensis* used in this study in China; Figure S3: The best AUC value of model prediction with ROC curves under current conditon; Figure S4: Correlation analysis between pairwise linearized Φ_ST_/(1 − Φ_ST_) values and the logarithm of geographic distance in Chinese populations of *Callosobruchus* *chinensis* based on concatenated mitochondrial genes; Figure S5: The results of the jackknife test on AUC for *Callosobruchus chinensis* in China to estimate environmental variable significance performed by Maxent; Table S1: Information on the 22 sites from which *C. chinensis* were collected; Table S2: Data on 80 occurrence points of *Callosobruchus chinensis* used in this study in China; Table S3: Indice of 19 environmental variables in Worldclim. References [102,103,104,105,106,107,108,109,110,111,112] are cited in the Supplementary Materials.
