# Supplementary material for: A Phylogeographical Analysis of the Beetle Pest Species Callosobruchus chinensis (Linnaeus, 1758) in China
Source: Insects. 2022 Jan 29;13(2):145. doi: 10.3390/insects13020145 (PMC8878040; doi:10.3390/insects13020145)

Insects

SUPPORTING INFORMATION

A Phylogeographical Analysis of the beetle pest species *Callosobruchus chinensis* (Linnaeus, 1758) in China

Fang Wang [1, †], Min Li[2, †], Haixia Zheng[1], Tian Dong[1] and Xianhong Zhang[1, *]

[1] College of Plant Protection, Shanxi Agricultural University, Jinzhong 030801, China 1; wangruifang24@163.com (F.W.); zhenghaixia722@163.com (H-X.Z.); 18234475698@163.com (T.D.)

[2] Department of Biology, Taiyuan Normal University, Jinzhong 030619, China 2; limin12nk@163.com (M.L.)

* Correspondence: Xianhong Zhang, College of Plant Protection, Shanxi Agricultural University, Jinzhong 030801, China. Email: zxh6288@126.com. Tel.: +86-1383-483-7840

† These authors contributed equally to this work.

Table S1. Information on the 22 sites from which *Callosobruchus chinensis* were collected.

Population Code	Sampling location	Longitude	Latitude
SJT	Taigu, Jinzhong, Shanxi	112.55°E	37.42°N
SCQ	Qin, Changzhi, Shanxi	112.7°E	36.75°N
SCW	Wuxiang, Changzhi, Shanxi	112.85°E	36.83°N
SLZ	Zhongyang, Lvliang, Shanxi	111.18°E	37.33°N
SXX	Xinfu, Xinzhou, Shanxi	112.73°E	38.42°N
SXH	Hequ, Xinzhou, Shanxi	111.13°E	39.38°N
SYQ	Yuanqu, Yuncheng, Shanxi	111.67°E	35.3°N
SSY	Youyu, Shuozhou, Shanxi	112.47°E	39.98°N
SD1	Hedong, Linyi, Shandong	118.4°E	35.08°N
SD2	Jinan, Shandong	116.98°E	36.67°N
HL	Luohe, Henan	114.02°E	33.58°N
HB1	Quzhou, Handan, Hebei	114.95°E	36.78°N
HB2	Neiqiu, Xingtai, Hebei	114.52°E	37.3°N
SW	Heyang, Weinan, Shaanxi	110.15°E	35.23°N
JX	Nanchang, Jiangxi	115.85°E	28.68°N
AH	Huaibei, Anhui	116.8°E	33.95°N
TJ	Tianjing	117.2°E	39.12°N
NM	Hohhot, Inner Mongolia	111.73°E	40.83°N
SC	Nanchong, Sichuan	106.08°E	30.78°N
HJ	Duodao, Jingmen, Hubei	112.2°E	30.98°N
JS	Xuanwu, Nanjing, Jiangsu	118.8°E	32.05°N
GZ	Fenggang, Zunyi, Guizhou	107.72°E	27.97°N

Figure S1. Occurrence Data from field collection

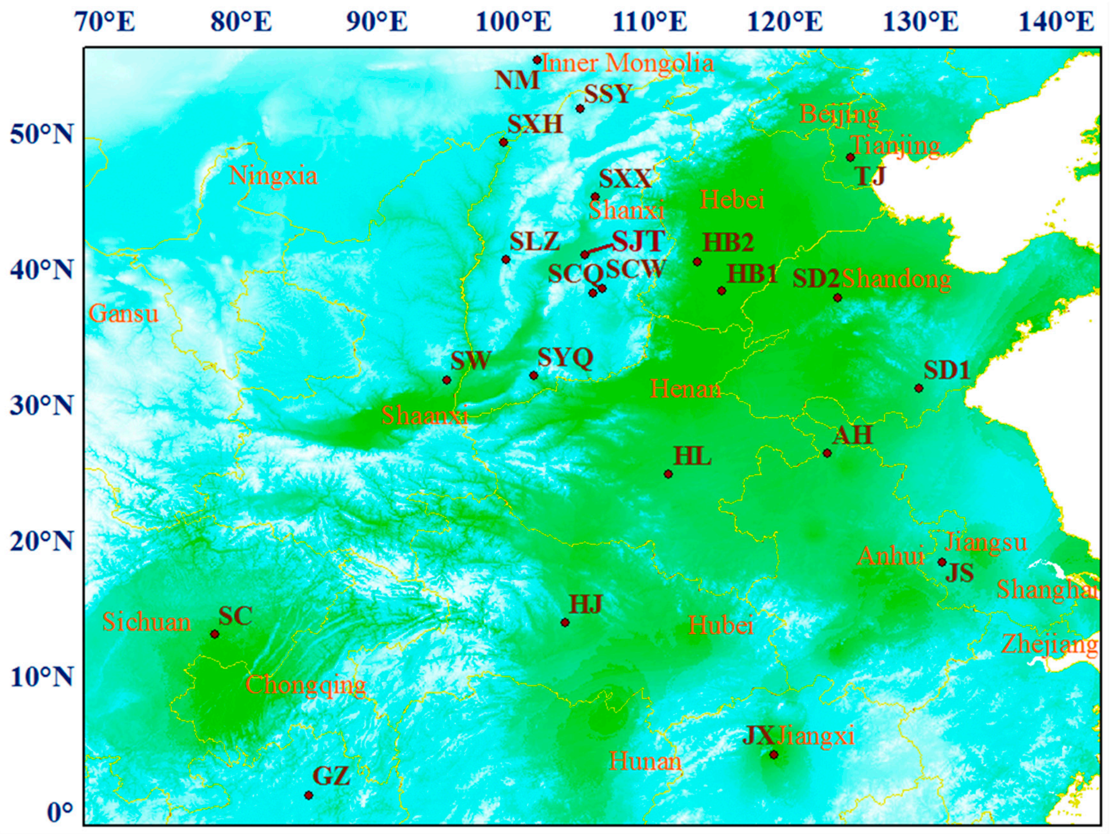

Table S2. Data on 80 occurrence points of *Callosobruchus chinensis* used in this study in China

Location	Longitude	Latitude	Source
Mentougou, Beijing	116.1°E	39.93°N	[44]
Qingfeng, Henan	113.65°E	34.73°N	[102]
Jingning, Zhejiang	119.63°E	27.98°N	[103]
Ili, Xinjiang	81.32°E	43.92°N	[104]
Urumqi, Xinjiang	87.62°E	43.82°N	[44]
Luliang, Yunnan	103.67°E	25.03°N	[44]
Dali, Yunnan	100.23°E	25.6°N	[105]
Qujing, Yunnan	103.8°E	25.5°N	[105]
Kaiyuan,Yunnan	103.27°E	23.72°N	[105]
Kunming, Yunnan	102.72°E	25.05°N	[105]
Huangnihe, Fuyuan,Yunnan	104.25°E	25.67°N	[106]
Jiangchuan,, Yunnan	102.75°E	24.28°N	[44]
Bobai, Guangxi	109.97°E	22.28°N	[44]
Fuchuan, Guangxi	111.27°E	24.83°N	[44]
Daxin, Guangxi	107.2°E	22.83°N	[44]
Lingchuan, Shanxi	113.27°E	35.78°N	[44]
Hefei, Anhui	117.25°E	31.83°N	[44]
Mengcheng, Anhui	116.57°E	33.27°N	[44]
Rudong, Jiangsu	121.18°E	32.32°N	[44]
Lishui, Jiangsu	119.02°E	31.65°N	[44]
Dengzhou, Henan	112.08°E	32.68°N	[44]
Xinye, Henan	112.35°E	32.52°N	[44]
Yangyuan, Hebei	114.17°E	40.12	[44]
Tangshan, Hebei	118.2°E	39.63°N	[44]
Baoding, Hebei	115.47°E	38.87°N	[44]
Qingdao, Shandong	120.38°E	36.07°N	[44]
Shenyang, Liaoning	123.43°E	41.8°N	[107]
Qingzhou, Shandong	118.47°E	36.68°N	[108]
Xichang, Sichuan	102.27°E	27.9°N	[107]
Ningnan, Sichuan	102.77°E	27.07°N	[109]
Ebian, Sichuan	103.27°E	29.23°N	[109]
Dazu County, Chongqing	105.72°E	29.7°N	[107]
Chongqing	106.55°E	29.57°N	[110]
Wulong, Chongqing	107.75°E	29.33°N	[44]
Qianjiang, Chongqing	108.77°E	29.53°N	[44]
Hechuan, Chongqing	106.28°E	29.97°N	[109]
Beibei,Chongqing	106.4°E	29.8°N	[111]
Baicheng City, Jilin	122.85°E	45.62°N	[112]
Wuhan City, Hubei	114.33°E	30.5°N	[108]
Nanyang, Henan	112.52°E	33°N	collection
Taigu, Shanxi	112.55°E	37.42°N	collection

Qin, Shanxi	112.7°E	36.75°N	collection
Wuxiang, Shanxi	112.85°E	36.83°N	collection
Zhongyang, Shanxi	111.18°E	37.33°N	collection
Xinfu, Shanxi	112.73°E	38.42°N	collection
Hequ, Shanxi	111.13°E	39.38°N	collection
Yuanqu, Shanxi	111.67°E	35.3°N	collection
Youyu, Shanxi	112.47°E	39.98°N	collection
Zhengwang, Shandong	118.4°E	35.08°N	collection
Jinan, Shandong	116.98°E	36.67°N	collection
Luohe City, Henan	114.02°E	33.58°N	collection
Quzhou, Hebei	114.95°E	36.78°N	collection
Neiqiu, Hebei	114.52°E	37.3°N	collection
Heyang, Shaanxi	110.15°E	35.23°N	collection
Huaibei City, Anhui	116.8°E	33.95°N	collection
Tianjin	117.2°E	39.12°N	collection
Hohhot in Inner Mongolia	111.73°E	40.83°N	collection
Nanchong City, Sichuan	106.08°E	30.78°N	collection
Macheng, Hubei	112.2°E	30.98°N	collection
Xuanwu, Jiangsu	118.8°E	32.05°N	collection
Fenggang, Guizhou	107.72°E	27.97°N	collection
Guangzhou, Guangdong	113.27°E	23.13°N	GBIF
Huiyang, Guangdong	114.47°E	22.8°N	GBIF
Zhongshan, Guangdong	113.38°E	22.52°N	GBIF
Lianzhou,Guangdong	112.38°E	24.78°N	GBIF
Huangmei, Hubei	115.93°E	30.08°N	GBIF
Haikou, Hainan	110.35°E	20.02°N	GBIF
Tainan, Taiwan	120.2°E	23°N	GBIF
Pingtung, Taiwan	120.48°E	22.67°N	[43]
Tainan, Taiwan	120.32°E	23.32°N	[43]
Danyang, Jiangsu	119.57°E	32°N	[108]
Wujiang, Jiangsu	120.63°E	31.17°N	[108]
Taizhou, Jiangsu	119.92°E	32.45°N	[108]
Xinjian, Jiangxi	115.82°E	28.7°N	[108]
Tai'an, Shandong	117.08°E	36.2°N	[108]
Yangling, Shaanxi	108.07°E	34.28°N	[108]
Tarim, Xinjiang	81.28°E	40.55°N	[108]
Wuhu City, Anhui	118.38°E	31.33°N	[108]
Jingzhou, Hubei	112.23°E	30.33°N	[108]
Minqin, Gansu	103.08°E	38.62°N	[108]

Figure S2. Data on 80 occurrence points of *Callosobruchus chinensis* used in this study in China.

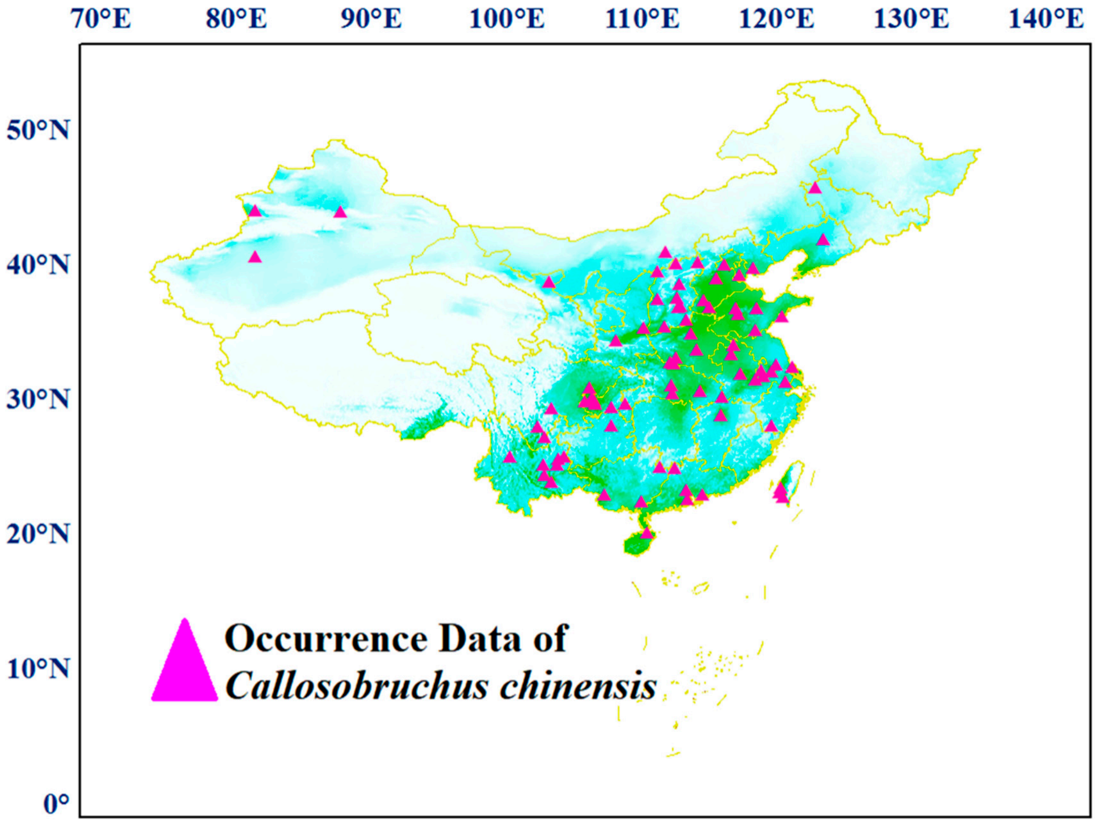

Table S3 Indice of 19 environmental variables in Worldclim.

Name of data	Description
Bio1	Annual mean temperature(°C)
Bio2	Mean diurnal range (mean of monthly [max temp-min temp])(°C)
Bio3	Isothermality(bio2/bio7) *100
Bio4	Temperature seasonality (standard deviation *100)
Bio5	Max temperature of warmest month(°C)
Bio6	Min temperature of coldest month(°C)
Bio7	Temperature annual range(bio5-bio6) (°C)
Bio8	Mean temperature of wettest quarter(°C)
Bio9	Mean temperature of driest quarter(°C)
Bio10	Mean temperature of warmest quarter(°C)
Bio11	Mean temperature of coldest quarter(°C)
Bio12	Annual precipitation(mm)
Bio13	Precipitation of wettest month(mm)
Bio14	Precipitation of driest month(mm）
Bio15	Precipitation seasonality (Coefficient of Variation)
Bio16	Precipitation of wettest quarter(mm)
Bio17	Precipitation of driest quarter（mm）
Bio18	Precipitation of warmest quarter（mm）
Bio19	Precipitation of coldest quarter（mm）

Figure S3. The best AUC value of model prediction with ROC curves under current conditon. The area under the curve (AUC) of receiver operating characteristic is to evaluate accuracy of model performance.

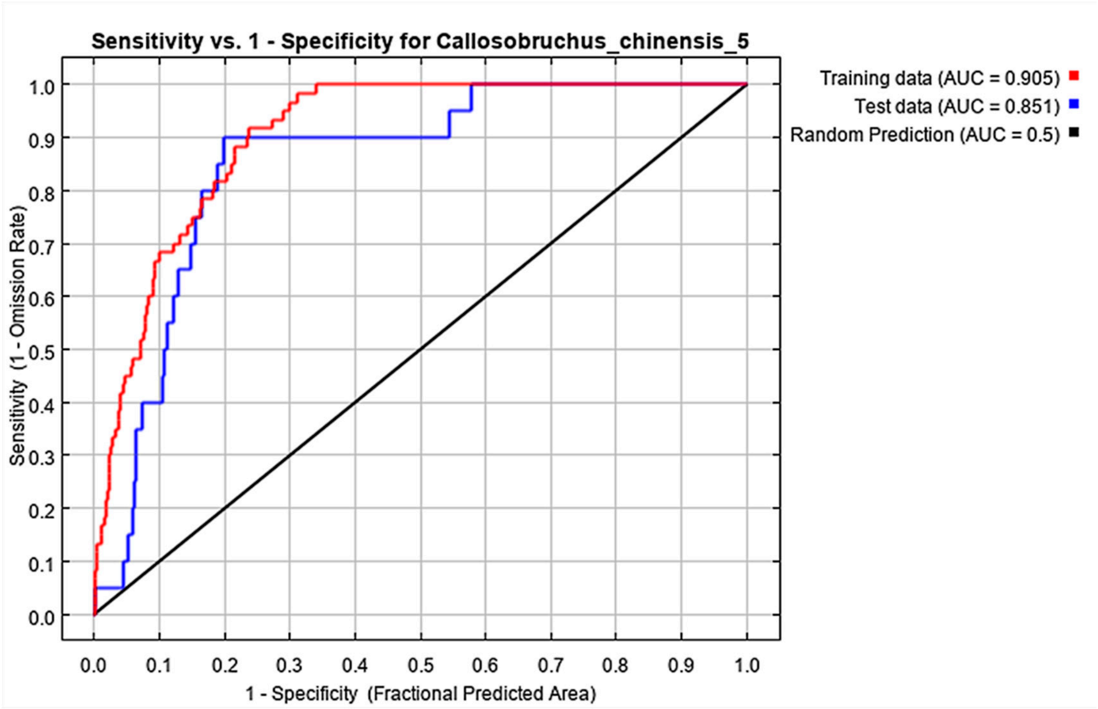

Figure S4. Correlation analysis between pairwise linearized Φ_{ST} / (1- Φ_{ST}) values and the logarithm of geographic distance in Chinese populations of *Callosobruchus chinensis* based on concatenated mitochondrial genes.

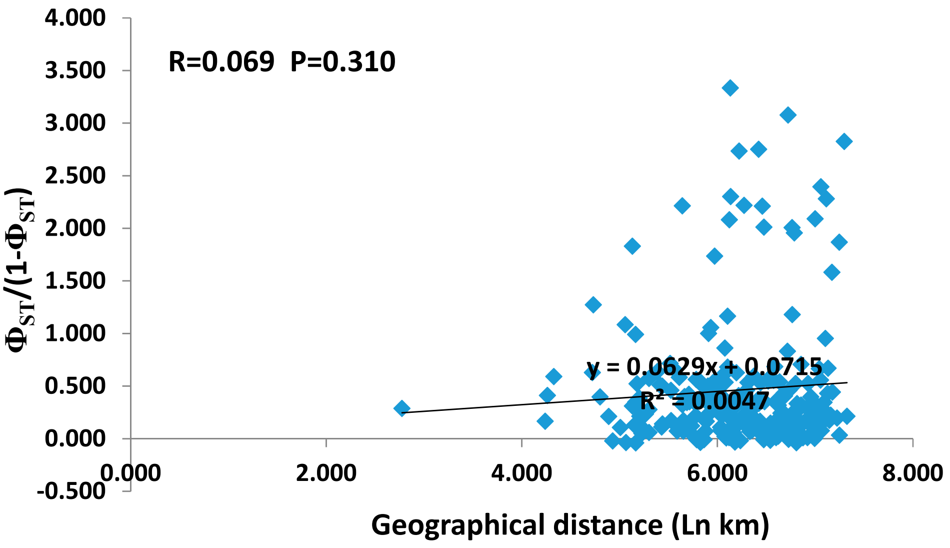

Figure S5. The results of the jackknife test on AUC for *Callosobruchus chinensis* in China to estimate environmental variable significance performed by Maxent.

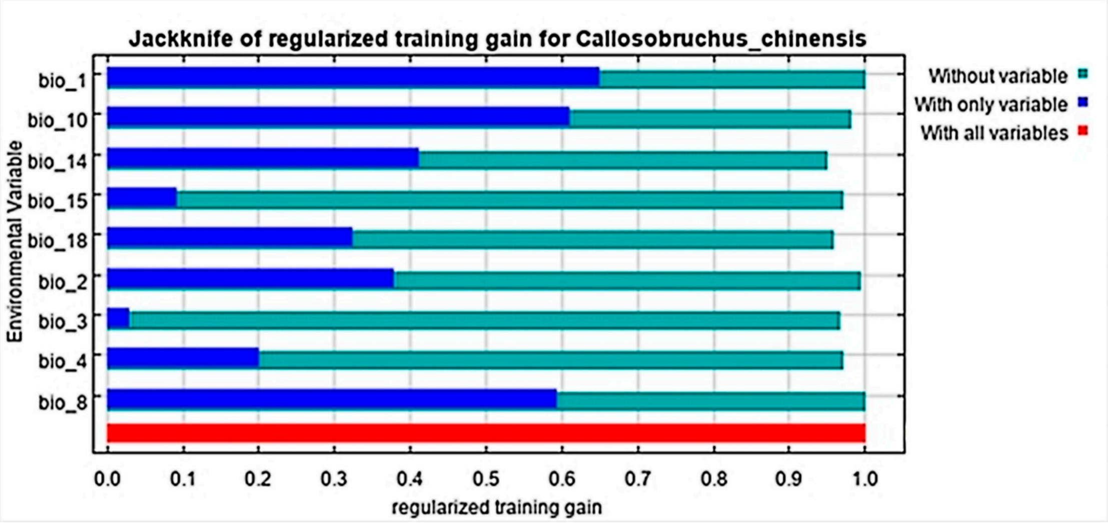

Supplement: Supplementary file 1 [file insects-13-00145-s001.zip › insects-1496106-supplementary.pdf]
